# Cardio-Metabolic Effects of High-Fat Diets and Their Underlying Mechanisms—A Narrative Review

**DOI:** 10.3390/nu12051505

**Published:** 2020-05-21

**Authors:** Jibran A. Wali, Natalia Jarzebska, David Raubenheimer, Stephen J. Simpson, Roman N. Rodionov, John F. O’Sullivan

**Affiliations:** 1 Charles Perkins Centre, The University of Sydney, Sydney, NSW 2006, Australia; david.raubenheimer@sydney.edu.au (D.R.); stephen.simpson@sydney.edu.au (S.J.S.); 2 Faculty of Science, School of Life and Environmental Sciences, The University of Sydney, Sydney, NSW 2006, Australia; 3 University Center for Vascular Medicine Department of Medicine III—Section Angiology, University Hospital Carl Gustav Carus, Technische Universität Dresden, 01307 Dresden, Germany; Natalia.Jarzebska@uniklinikum-dresden.de (N.J.); Roman.Rodionov@uniklinikum-dresden.de (R.N.R.); 4 Department of Anesthesiology and Intensive Care Medicine, University Hospital Carl Gustav Carus, Technische Universität Dresden, 01307 Dresden, Germany; 5 Faculty of Medical Sciences, School of Medicine, The University of Sydney, Sydney, NSW 2006, Australia; 6 Heart Research Institute, The University of Sydney, Sydney, NSW 2006, Australia; 7 Department of Cardiology, Royal Prince Alfred Hospital, Camperdown, NSW 2050, Australia

**Keywords:** high-fat diet, saturated fatty acids, metabolism, insulin resistance, obesity, diabetes, mice, cardiovascular disease

## Abstract

The majority of the epidemiological evidence over the past few decades has linked high intake of fats, especially saturated fats, to increased risk of diabetes and cardiovascular disease. However, findings of some recent studies (e.g., the PURE study) have contested this association. High saturated fat diets (HFD) have been widely used in rodent research to study the mechanism of insulin resistance and metabolic syndrome. Two separate but somewhat overlapping models—the diacylglycerol (DAG) model and the ceramide model—have emerged to explain the development of insulin resistance. Studies have shown that lipid deposition in tissues such as muscle and liver inhibit insulin signaling via the toxic molecules DAG and ceramide. DAGs activate protein kinase C that inhibit insulin-PI3K-Akt signaling by phosphorylating serine residues on insulin receptor substrate (IRS). Ceramides are sphingolipids with variable acyl group chain length and activate protein phosphatase 2A that dephosphorylates Akt to block insulin signaling. In adipose tissue, obesity leads to infiltration of macrophages that secrete pro-inflammatory cytokines that inhibit insulin signaling by phosphorylating serine residues of IRS proteins. For cardiovascular disease, studies in humans in the 1950s and 1960s linked high saturated fat intake with atherosclerosis and coronary artery disease. More recently, trials involving Mediterranean diet (e.g., PREDIMED study) have indicated that healthy monounsaturated fats are more effective in preventing cardiovascular mortality and coronary artery disease than are low-fat, low-cholesterol diets. Antioxidant and anti-inflammatory effects of Mediterranean diets are potential mediators of these benefits.

## 1. Introduction

More than half of the adult population in Europe and two-thirds in the United States are either overweight or obese [1,2]. Around 641 million adults worldwide are obese, and more than 450 million adults are diabetic [3,4]. Wide-ranging factors including intra-uterine and postnatal growth, genetic susceptibility, unhealthy diet, socioeconomic status, physical activity and hormonal disorders contribute to obesity and related metabolic disorders [5]. Out of these, “unhealthy” diet is the major modifiable factor that can be targeted to control obesity and metabolic disease. But despite several recommendations and guidelines for “healthy” diets with different macronutrient compositions, a substantial reduction in body weight, and long-term compliance across populations have not been achieved so far [6].

For several decades, because of their greater caloric density, dietary fats have been described as an integral ingredient of an “unhealthy” diet. Increased fat consumption has been considered responsible for obesity and associated metabolic disease [7,8]. In particular, saturated fats have been linked to adverse cardiovascular outcomes [9,10]. Diets rich in saturated fats have been widely employed in rodent studies to study the mechanisms of insulin resistance [11,12]. In this narrative review, we discuss the epidemiological and experimental evidence linking fats to cardio-metabolic disease. We also provide an in-depth overview of the findings from animal studies that have led to ceramide and diacylglycerol models of insulin resistance.

## 2. Epidemiological Evidence for the Association Between Dietary Fat and the Risk of Obesity and Cardiovascular Disease

The prevalence of obesity and type 2 diabetes has dramatically increased over the past few decades worldwide [13]. It is widely accepted that increased intake of fat, especially saturated fat, is a major driver of the increase in obesity and increased incidence of cardiometabolic disease [7,8]. For example, analysis of data from 20 countries showed that the proportion of overweight/obese subjects positively correlated with the proportion of energy intake from fat [14]. In addition, analysis of data from 28 trials showed a decrease in body weight of 1.6 g/d for every percentage decrease in daily energy intake from fat [14]. Specifically, increased consumption of saturated fats has been associated with risk factors such as elevated concentrations of low-density lipoprotein (LDL)-cholesterol and apolipoprotein B [9,10,15]. Similarly, high intake of trans unsaturated fatty acids increases the risk of heart disease and cardiovascular mortality [9,16]. Current dietary recommendations advocate restricting the consumption of saturated fatty acids for cardiovascular health benefits [9]. In contrast, a meta-analysis of observational studies and clinical trial showed that saturated fat consumption is not associated with increased risk of coronary artery disease [17]. Similarly, another meta-analysis showed that saturated fat intake is not associated with coronary artery disease or stroke [18]. This has led to calls for revising the guidelines against decreasing saturated fat intake. Although there is disagreement on the role of reducing saturated fat consumption for cardiovascular health, consensus is emerging that instead of focusing on individual nutrient (saturated fat) the recommendations should be food-based. Furthermore, not all types of fats are equal in their effects, and evidence suggests that replacing dietary saturated fatty acids with polyunsaturated fatty acids (PUFA) reduces the risk of cardiovascular mortality [9,19]. These PUFAs are of two main types: Omega-6 PUFAs are found in vegetables and vegetable oils; omega-3 PUFAs include alpha-linolenic acid (ALA), eicosapentaenoic acid (EPA), and docosahexaenoic acid (DHA). ALA is sourced from vegetable oils while seafood including fish and shellfish is rich in EPA and DHA [9,19].

## 3. Fat vs. Carbohydrate Intake and Cardiometabolic Risk

A consequence of the recommendations to cut down saturated fat intake has been a concomitant increase in the proportion of refined carbohydrates in the diet [20]. Therefore unsurprisingly, the focus in recent years has gradually shifted from saturated fat intake to carbohydrate intake, with particular interest in the relationship between processed carbohydrates including fructose-containing sugars and obesity [21]. This has led to controversy and the “fat vs. carb” debate in the nutrition science community [22]. Most notably, the Prospective Urban Rural Epidemiology (PURE) study—one of the most extensive studies in this field in recent years—has suggested that fat intake does not increase the risk of cardiometabolic disease. This study documented macronutrient consumption data of 100,000 adults between 2003 and 2013 across 18 countries around the world [23]. The results showed that dietary fat intake reduced the risk of all-cause mortality (Hazard Ratio 0.77 for quintile 5 vs. quintile 1). Similarly, comparison of highest vs. lowest quintile showed a decrease in total mortality with increasing consumption of saturated fats [23]. However, the risk of mortality increased with carbohydrate intake (HR 1.28) [23]. Isocaloric (5% energy) substitution of carbohydrate with saturated fatty acids reduced the risk of stroke by 20% [23]. Fat intake was associated with improvements in lipid markers of cardiovascular health (lower ratios of total cholesterol and high-density lipoprotein (HDL) cholesterol, lower triglyceridemia and lower ratio of apolipoprotein B to ApoA1), but these markers became worse with carbohydrate intake [24]. Therefore, it was proposed that current dietary guidelines should be modified to endorse the benefits of fat intake [23]. However, the authors of the PURE study have acknowledged that the food frequency questionnaires used in their work are not a reliable measure of absolute intake, and dietary intake assessments only at baseline is a major limitations of their work [23,24]. Others have questioned the accuracy of the PURE study data for carbohydrate and fat intake for a Chinese population [25]. In addition, Seidelmann et al., have shown that the relationship between carbohydrate consumption and mortality risk is U-shaped, the mortality risk being lowest when 50–55% of calories are sourced from carbohydrates. The increase in mortality risk reported by the PURE study is based on the data comparing very high carbohydrate intake (70% energy) with moderate carbohydrate intake (45–55% energy) [26]. Replacing carbohydrates with plant-based fat and protein was found to reduce the risk of mortality, but animal-derived fat and protein had opposite effects [26]. These observations underscore the importance of a multi-nutrient/food-based approach to nutrition science research to address the disagreements about the role of fat in causing metabolic disease [27].

## 4. Role of Dietary Fat in Regulating Food and Caloric Intake

While carnivore animals prioritize fat intake, dietary studies have shown that omnivorous species including mice and humans prioritize protein intake over carbohydrate and fat [28,29,30,31,32,33]. When the concentration of dietary protein decreases because of its dilution with fat and/or carbohydrate, these animals overconsume such a diet in an effort to reach their protein “intake target”. This results in increased caloric intake on protein-diluted diets, a phenomenon known as “protein leverage” [34]. Conversely, diets high in protein content lead to reduced caloric intake as the intake target for protein is reached with lower total food intake [31,34]. Mice fed 25 different isocaloric diets containing different ratios of protein, fat, and carbohydrate showed that the animals regulated their protein intake more tightly than that of carbohydrate or fat, increasing food intake on low protein diets [30]. Regulatory feeding was also seen for carbohydrate, but was less marked than for protein [30]. On the contrary, dietary fat content had minimal effect on food intake and fat intake was relatively unregulated [30]. In contrast, carnivorous animals such as spiders, predatory beetles, cats, and mink give priority to fat intake (even over protein intake) and adjust their total food consumption to reach their fat intake target when provided diets with different amounts of fat [32,33,35,36].

Dietary fat can induce obesity because of its palatability, especially when combined in foods with sugar and salt, and high caloric content. The energy density of fat (9 kcal/g) is ca. double that of carbohydrate (4 kcal/g) and protein (4 kcal/g) [6,22,37]. Therefore, replacing carbohydrate with fat on a gram for gram basis will increase the energy density of the food item [6,22,37]. Consequently, in humans, energy intake will tend to be elevated on diets that are low in protein, high in fat, sugar and salt, and low in fiber [30]. This combination is often found in ultra-processed food items, and increased consumption of these foods leads to weight gain and obesity [38,39].

## 5. High-Fat Diet Rodent Model

Rodents maintained on high-fat diets (HFD) are commonly used in research to model human metabolic syndrome. In particular, Wistar/Sprague-Dawley rats and C57BL/6 mice fed HFD have been extensively used as a model of diet-induced obesity (DIO) to study the mechanisms of insulin resistance as they are comparatively more susceptible to metabolic impairment [40]. For example, the C57BL/6 strain is more prone to develop obesity and insulin resistance than A/J, C57BLKS/J, BALB/c, FVB/N, and 129S6 mice [40,41,42,43]. The DIO rodent models are more relevant to humans than the monogenic animal models of obesity (*fa*/*fa* Zucker fatty rats, *ob*/*ob* and *db*/*db* mice) as germline defects in leptin production/signaling are rare in humans [43]. The two commonly used sub-strains of C57BL/6 mice used in DIO studies are C57BL/6J (from JAX lab) and C5BL/6N (from NIH) [41]. The C57BL/6J mice contain a mutation in the nicotinamide nucleotide transhydrogenase (*Nnt*) gene, gain more body weight, and have higher blood glucose levels and glucose tolerance than C57BL/6N mice on HFD [41,44]. The HFDs used in metabolic research typically provide 40–60% of calories from fat, and the commonly used sources of fat include lard and beef tallow that are rich in saturated fatty acids [40]. In addition, plant oils including corn and safflower oil have also been used in HFDs [40]. The energy density of these diets is often higher than control diets used in rodent studies, which contributes to making them obesogenic [45]. Male mice are mostly used in experiments as they are more prone to HFD-induced insulin resistance than females [46].

**Figure 1:**
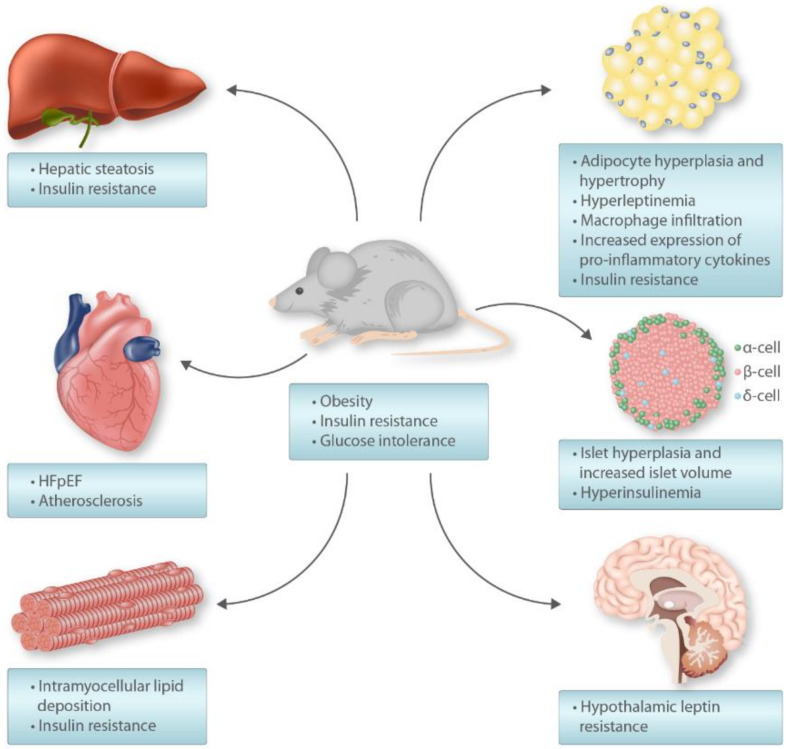
Cardiometabolic phenotype of HFD-fed mice. HFpEF: Heart failure with preserved ejection fraction.

Compared with standard chow-fed control mice, HFD induces obesity, moderate hyperglycemia, hyperinsulinemia, hypertriglyceridemia, steatosis, beta-cell dysfunction and hypertrophy, and insulin resistance in muscle and liver (Figure 1) [40,42]. The obesity involves both adipocyte hyperplasia and hypertrophy [42]. These diets induce glucose intolerance and hepatic insulin resistance in mice within a week [43,47]. However, other features of the metabolic phenotype usually become apparent after more than 4 weeks of feeding [40,42]. In many cases, HFD are also high in their sucrose (disaccharide of glucose and fructose) content, which is added to model fat and sugar-rich western diets [48]. This is because fructose potently stimulates de novo lipogenesis (DNL) in the liver and promotes visceral obesity [49]. Therefore, the addition of sucrose to a HFD (high-fat–high-sucrose diet; HFHSD) exacerbates the obesity and insulin resistance phenotype [50,51]. Contrary to the fatty liver induced by HFD feeding, mice fed HFHSD additionally show signs of low-grade inflammation and fibrosis [48]. Thus, pure HFD does not lead to non-alcoholic steatohepatitis (NASH) in mice and addition of excess cholesterol and fructose to a HFD is required for hepatocyte ballooning and fibrosis [52]. Interestingly, when fed for long-term (12 months), the glucose tolerance of HFHSD-fed mice becomes similar to standard chow-fed control mice [47]. This is caused by a compensatory expansion of pancreatic beta-cells mass and consequent hyperinsulinemia [47]. Furthermore, it is important to note that HFD and HFHSD induce only moderate hyperglycemia, and these dietary interventions do not lead to overt diabetes [40,42].

## 6. High-Fat Diet-Induced Insulin Resistance

Mice and rats made obese by HFD-feeding have been widely used in experimental studies to investigate insulin signaling and insulin resistance at the molecular level. Insulin receptors are ubiquitously distributed in mammalian cells but major sites of insulin action include hepatocytes, adipocytes, skeletal muscle cells, and neurons [11,53]. In the liver, insulin inhibits gluconeogenesis, stimulates glycogen synthesis and lipogenesis. Insulin signaling in skeletal muscle leads to glucose uptake and protein synthesis, while in adipose tissue insulin inhibits lipolysis and promotes glucose and fatty acid uptake [53]. Insulin-induced glucose uptake by muscle and fat cells involves translocation of glucose transporter type 4 (GLUT4) to the cell membrane. In neuronal cells, insulin activates locomotor and satiety signals [11,53].

### 6.1. Insulin Signaling Pathway

Binding of insulin to its receptor leads to phosphorylation of tyrosine residues in the receptor (IR) itself and in the insulin receptor substrates (mainly IRS1 and IRS2) [53]. Phosphorylation of IRS results in downstream activation of phosphatidylinositol 3-kinase (PI3K) and subsequent synthesis of triphosphorylated inositol (PIP_3_) at the cell membrane [54]. This is followed by the recruitment of phosphoinositide-dependent kinase (PDK), which activates Akt—a serine/threonine kinase—by phosphorylating it [54]. This activation of Akt links insulin signaling with translocation of GLUT4 to cell membrane in skeletal muscle and fat cells, glycogenesis, mammalian target of rapamycin (mTOR) activation and inhibitory phosphorylation of type 1 of the forkhead transcription factors of the O class (FOXO1) [53,54]. In general, IR and IRS proteins are activated by tyrosine phosphorylation and inhibited by phosphorylation of their serine or threonine residues [53].

Insulin signaling in the liver inhibits gluconeogenesis by phosphorylation of FOXO1 and increases lipogenesis by activating transcription factor sterol regulatory element-binding protein (SREBP)-1c [11,43]. FOXO1 is a transcription factor that induces the expression of genes associated with gluconeogenesis such as phosphoenolpyruvate carboxykinase (*PEPCK*) and glucose 6-phosphatase (*G6Pase*) [11]. Insulin-mediated phosphorylation of FOXO1 prevents it from entering the nucleus which decreases *PEPCK* and *G6Pase* transcript abundance [55]. SREBP1c is a transcription factor that increases the expression of genes involved in triglyceride synthesis including acetyl-CoA-caboxylase (*ACC*) and fatty acid synthase (*FAS*) [53]. Insulin activates SREBP1c by increasing its gene expression as well as acting post-translationally by increasing its processing to the mature form that translocates to the nucleus [55].

**Figure 2:**
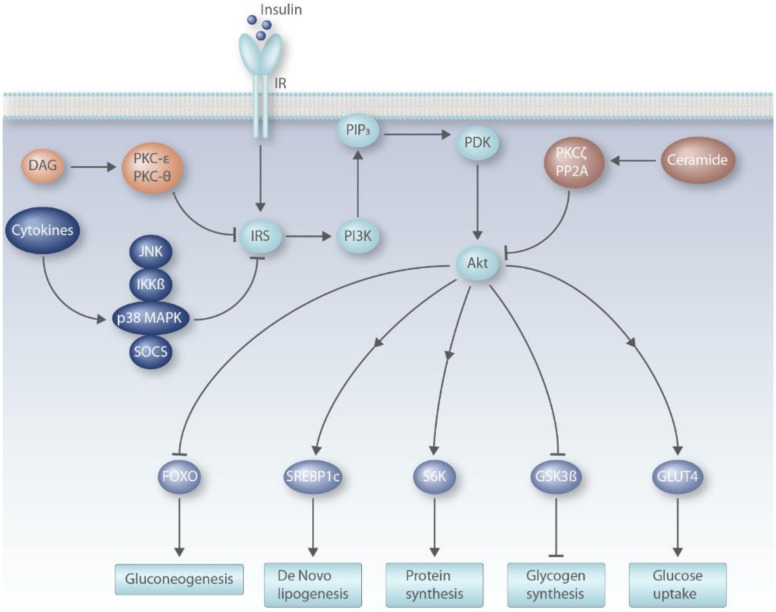
Molecular mechanisms of insulin actions and insulin resistance. DAG: diacylglycerol; FOXO: Forkhead box protein O; GLUT4: glucose transporter type 4; GSK3ß: glycogen synthase kinase 3 beta; IKKß: inhibitor of nuclear factor kappa-B kinase subunit beta; IR: insulin receptor; IRS: insulin receptor substrate; JNK: c-Jun N-terminal kinases; MAPK: mitogen-activated protein kinase; PDK: phosphoinositide-dependent kinase; PI3K: phosphoinositide 3-kinases; PIP_3_: phosphatidylinositol (3,4,5)-trisphosphate; PKC: protein kinase C; PP2A: protein phosphatase 2A; S6K: ribosomal protein S6 kinase; SOCS: suppressor of cytokine signaling; SREBP1: sterol regulatory element-binding protein 1.

Insulin resistance is characterized by impaired insulin actions in target tissues and includes reduced glucose uptake in fat and muscle tissues, decreased suppression of endogenous glucose production in the liver, and reduced suppression of lipolysis in adipose tissue and a reduction in insulin-induced glycogen synthesis [11,12]. Mechanistically, ceramides, diacylglycerol, and pro-inflammatory cytokines have been described in the literature as possible mediators of insulin resistance (Figure 2) [11,12].

### 6.2. DAG Model of Insulin Resistance

High-fat feeding in rodents increases the levels of sn-1,2-diacylglycerol (DAG) in liver and muscle and accumulation of DAG in the tissues activates calcium-independent “novel” isoforms of the protein kinase C (PKC) family [12]. In the liver, DAGs activate PKC-ε while in muscle PKC-θ is activated [54]. PKC activation results in phosphorylation of serine residues on IRS1 which blocks insulin-stimulated activating phosphorylation of tyrosine residues and decreases insulin-PI3K-Akt signaling [56]. In skeletal muscle, the decrease in insulin-PI3K-Akt signaling results in reduced GLUT-4 mediated glucose uptake in skeletal muscle tissue [11,43].

The role of DAG mediated PKC-ε activation in the context of hepatic insulin resistance has been well studied. It has been shown that DAG-mediated activation of PKC-ε inhibits hepatic insulin signaling by phosphorylation of Thr1160 in the activation loop of IR that blocks its kinase activity [57]. Supporting the DAG model of insulin resistance, in obese subjects with non-alcoholic fatty liver disease (NAFLD), hepatic DAG content negatively correlated with insulin-induced suppression of hepatic glucose production [58]. Furthermore, hepatocyte cytosolic DAG content was associated with PKC-ε translocation from cytosol to plasma membrane, and the compartmentalization of DAG in plasma membrane was found to be critical in activating PKC-ε and inducing insulin resistance [12,59,60]. Comparative gene identification (CGI)-58 is a lipid droplet associated protein that regulates triglyceride lipase activity. In control HFD-fed mice, DAG increased in membrane fraction, but in CGI-58 knockdown mice, the increase in DAG was mainly in lipid droplet and endoplasmic reticulum (ER) that did not cause PKC-ε activation [60].

The DAG-PKC-ε model of hepatic insulin resistance has been challenged in a recent study. Global PKC-ε deletion protected HFD-fed mice from insulin resistance and glucose intolerance [61,62]. In contrast, liver specific deletion of PKC-ε did not protect HFD-fed mice from glucose intolerance or insulin resistance (assessed by clamp studies) [61]. Interestingly, loss of PKC-ε in adipose tissue improved glucose tolerance but did not affect insulin-stimulated suppression of hepatic glucose production in HFD-fed mice [61]. Overall, this work showed that regulation of glucose homeostasis by PKC-ε is not mediated by its direct effects in the liver [61]. Loss of PKC-ε in adipocytes affected the expression of several genes associated with hepatocyte growth and metabolism suggesting that PKC-ε mediates metabolic cross talk between liver and adipose tissue [61].

It is not entirely clear why the findings of Brandon et al. [61] were contrary to previous findings of Shulman and colleagues that linked DAG-induced PKC-ε activation to hepatic insulin resistance [57]. It is possible that effects of hepatic PKC-ε deletion by Cre-Lox system were obscured by adaptive changes in the liver [63]. Brandon et al., mainly used euglycemic clamps to assess tissue specific insulin signaling [61]. Future studies should examine the effects of acute PKC-ε in adult animals and complement clamp studies with more direct assessment of hepatic insulin signaling by determining insulin-induced phosphorylation of Akt and IRS proteins.

### 6.3. Ceramide Model of Insulin Resistance

Ceramides are structural components of plasma membranes and are sphingolipids consisting of a sphingosine backbone joined to a fatty acid of variable length via an amide linkage [64]. The rate-limiting step in their biosynthesis is catalyzed by the enzyme serine palmitoyltransferase (SPT) [64,65]. Rats treated with dexamethasone (glucocorticoid) showed increased ceramide content in the liver and elevated hepatic SPT expression. Pre-treatment of rats with myriocin (SPT inhibitor) protected from dexamethasone induced insulin resistance and glucose intolerance [65]. Similarly, rats administered lard-oil infusion (rich in saturated fatty acid palmitate) showed an increase in serum free fatty acid levels and ceramide abundance in liver and skeletal muscle [65]. Hyperinsulinemic-euglycemic clamp studies showed that lard infusion caused insulin resistance in liver and muscle, but these effects were reduced by myriocin [65]. In contrast, insulin resistance induced by soy oil infusion (rich in unsaturated fatty acid linoleate) was not inhibited by myriocin [65]. Similarly, in C2C12 myotubes, treatment with long chain saturated fatty acids (palmitate, stearate, arachidate, lignocerate) induced ceramide accumulation and inhibited insulin signaling, but these effects were not reproduced with unsaturated fatty acid oleate [66]. These studies showed that saturated fats, but not unsaturated fats, inhibit insulin signaling in a ceramide dependent manner [65].

The chain length of the acyl group in ceramides ranges from C_14:0_ to C_30:0_ and this is dependent on the ceramide synthase enzyme (CerS1-6) involved in their biosynthesis [67]. In obese humans and HFD-fed mice, abundance of C_16:0_ ceramide and CerS6 expression is increased in adipose tissue, and the latter was demonstrated to be significantly correlated with insulin resistance in humans [67]. Mice deficient in CerS6 were protected from HFD-induced obesity, showed improvements in glucose tolerance and insulin sensitivity, and increased energy expenditure coupled with higher fat oxidation in brown adipose tissue and liver [67]. This was associated with increased insulin stimulated Akt phosphorylation in liver, but not in skeletal muscle [67]. Ceramides contain a 4,5-trans double bond in their sphingoid base, but this bond is absent in dihydroceramides [68]. The ER resident enzyme dihydroceramide desaturase (Des1) inserts this double bond into ceramides [68]. Deletion of gene encoding Des1 resulted in increased ratio of dihydroceramide/ceramide in various tissues [68]. Obese mice (*ob*/*ob* and HFD) lacking Des1 were protected from obesity, fatty liver, and insulin resistance [68]. In hepatocytes, ceramide inhibited insulin-induced Akt phosphorylation, and loss of Des1 reduced lipogenesis and increased mitochondrial activity [68]. This showed that the presence of double bond is essential for ceramide-induced impairment of glucose homeostasis. Thus, DES1 is a potential therapeutic target for fatty liver, insulin resistance, and associated metabolic disorders.

At a molecular level, ceramide induces dephosphorylation of Akt by activating protein phosphatase 2A, and this leads to inhibition of insulin signaling [69]. In addition, the translocation of Akt to cell membrane is also blocked by ceramide [69,70]. This is mediated by an inhibitory phosphorylation of Akt via PKCζ activation [70]. Contrary to these observations, some investigators have suggested that effects of ceramides on insulin signaling are indirectly mediated by changes in mitochondrial function [71]. For example, myriocin has been shown to improve mitochondrial electron transport chain activity and fatty acid oxidation [72] which could contribute to the observed improvements in insulin sensitivity [65]. Further, while the hepatic abundance of C_16:0_ ceramide is reduced in CerS6 as well as CerS5 knockout mice, only CerS6 deficient mice are protected from HFD-fed obesity, hepatic steatosis, glucose intolerance, and insulin resistance [73]. Hepatic mitochondria lacking CerS6, but not CerS5, showed increased mitochondrial activity [73]. This is because only those C_16:0_ sphingolipids that are synthesized by CerS6 interact with the mitochondrial fission-associated factor Mff, and this interaction is a mediator of the increase in mitochondrial fragmentation caused by HFD-induced obesity [73]. Overexpression of CerS6 in the liver of mice increased C_16:0_ ceramide content, impaired glucose homeostasis, and altered mitochondrial morphology. However, these effects were abrogated by concomitant Mff knockdown [73]. Thus, the increase in C_16:0_ ceramide does not lead to metabolic impairment in the absence of attendant mitochondrial dysfunction, and this dysfunction could decrease fat oxidation resulting in DAG accumulation, ultimately leading to reduced insulin signal transduction [12].

### 6.4. Pro-Inflammatory Cytokines

HFD and obesity result in adipocyte hypoxia that ultimately results in adipocyte cell death [12]. This causes macrophage recruitment and secretion of pro-inflammatory cytokines [12,74]. Specifically, an increase in classically activated pro-inflammatory M1 macrophages and effector T cells in adipose tissue is observed in obesity and insulin resistance in mice and humans [74,75,76]. There is also a decrease in alternatively activated anti-inflammatory M2 macrophages and regulatory T cells [54,75]. The M1 macrophages infiltrating the adipose tissue secrete pro-inflammatory cytokines tumor necrosis factor (TNF)α, interleukin (IL)-6 and IL-1ß [53]. In addition to local effects in adipose tissue, these cytokines are transported to the liver and muscle via systemic circulation where they reduce insulin sensitivity [74]. The role of these cytokines in regulating glucose homeostasis was first described in detail for TNFα [74]. Hotamisligil et al., showed an increase in adipose tissue TNFα expression and plasma TNFα levels in rodent models of obesity [77]. Obese mice lacking TNFα showed improved insulin sensitivity in metabolic tissues [78]. Compared with subcutaneous fat, the infiltration of macrophages is greater in visceral fat and is associated with relatively higher expression of cytokines [79]. The adipocyte-derived chemokines monocyte chemoattractant protein (MCP)-1 and leukotriene B4 (LTB4) are the major mediators of macrophage migration to adipose tissue [74]. In addition to adipose tissue, macrophage recruitment to liver and activation of Kupffer cells has also been linked to hepatic insulin resistance [80,81]. The pro-inflammatory cytokines induce insulin resistance by phosphorylating serine residues of IRS proteins. This is mediated by downstream activation of kinases such as inhibitor of nuclear factor kappa-B kinase subunit bet (IKKß), c-Jun N-terminal kinase (JNK)1, and p38 mitogen-activated protein kinases (MAPK) [82,83]. In addition, the pro-inflammatory cytokines increase the production of suppressor of cytokine signaling proteins (SOCS) proteins (SOCS-1, 3, 6, and 7) that block insulin signaling by blocking the tyrosine phosphorylation site of IRS proteins and increasing proteasomal degradation of IRS [54,84]. Through these mechanisms, tissue inflammation and circulating inflammatory cytokines contribute to systemic insulin resistance [53,74]. In addition, insulin resistance and associated inflammation stimulates lipolysis in adipose tissue which increases the supply of fatty acids and glycerol to the liver. These substrates fuel increased gluconeogenesis and induces insulin-independent de novo lipogenesis and ectopic lipid deposition in the liver [12]. Consequently, this leads to steatosis and worsening of hepatic insulin resistance [12].

Importantly, studies in rats have shown that ectopic lipid deposition in liver and muscle and insulin resistance in white adipose tissue develops within days of HFD-feeding, but adipocyte inflammation manifests itself after about four weeks, with infiltration of CD8 T cells taking place before macrophage accretion [76,85]. Thus, adipose tissue inflammation and increased circulating cytokine concentrations are long-term consequences of HFD-induced obesity that are important for progression of insulin resistance and systemic metabolic abnormalities [12]. In addition, despite the link between systemic low-grade inflammation and type 2 diabetes, there are currently no approved drugs for managing diabetes that work via modulating immune mediators [86]. Preliminary trials involving IL-1ß antagonism (anakinra and canakinumab), TNFα inhibitors (e.g., etanercept), NF-kB pathway inhibition (salsalate) have either shown limited long-term success in improving clinical outcomes or have been associated with safety issues or both [86,87,88,89,90,91]. However, a safe and effective drug for managing diabetes via immune modulation will have the likely added advantage of beneficial effects on cardiovascular complications and rheumatological disorders [86].

### 6.5. Selective Hepatic Insulin Resistance

Mouse models of obesity and insulin resistance as well as humans with type 2 diabetes show a combination of hyperglycemia, hyperinsulinemia, and hypertriglyceridemia [55]. Failure of insulin to inhibit hepatic glucose output and gluconeogenesis is the hallmark of hepatic insulin resistance [43]. Interestingly, in hepatic insulin resistance, the inhibition of glucose production by insulin is impaired but insulin-induced lipogenesis is unaffected. Insulin-stimulated phosphorylation of FOXO1 is reduced, expression of *PEPCK* and *G6Pase* genes remains elevated, but the lipogenic SREBP1c pathway remains sensitive to insulin, nuclear SREBP1c levels increase with hyperinsulinemia and this worsens triglyceridemia [55,92]. This phenomenon has been described as “selective insulin resistance” in the liver [55]. In contrast, examination of the metabolic phenotype of the mice with liver-specific deletion of insulin receptor (LIRKO mice) showed that these mice with total hepatic insulin resistance have hyperglycemia and hyperinsulinemia but their plasma triglyceride levels are lower than controls [93].

The concept of selective insulin resistance in liver derives from the assumption that hepatic triglyceride content is mainly regulated by DNL [12]. However, increased re-esterification of fatty acids supplied to the liver from augmented adipose tissue lipolysis also contributes significantly to the liver triglyceride abundance, and this is independent of hepatic insulin signaling [12,94]. In addition, hepatic DNL can be activated independently of insulin signaling by mechanisms such as activation of carbohydrate-responsive element-binding protein (ChREBP) and fructose induced DNL [12,95,96].

## 7. High-Fat Diet and the Heart

The fact that nutrition influences atherosclerosis has been evident since the first observation by Ignatowski [97] that diets high in animal proteins (milk, meat, and eggs) fed to rabbits caused intimal lesions with large clear cell (now called foam cell) accumulation in the aorta. Later, Anitschkow fed rabbits high cholesterol diets that produced aortic atherosclerosis similar to that seen in humans [98], and proposed a causal role of cholesterol in atherosclerosis, which remains the consensus [98]. Subsequently, cholesterol was isolated in human atherosclerotic plaques [99].

Epidemiological work in the 1950s found that indigenous diets were associated with high variability of coronary artery disease prevalence across different populations [100]. Individuals with coronary artery disease tended to have higher circulating cholesterol compared to their healthy counterparts. The Seven Countries study in 1957 found that populations with higher saturated fat intake had higher serum cholesterol, and follow-up work confirmed that they also had a greater burden of coronary artery disease [101]. This spurred a plethora of studies that examined whether reduction of saturated fat in the diet could reduce incidence of coronary artery disease. In 1968, the US National Diet-Heart Study showed that a low saturated fat could reduce serum cholesterol by 11–12% in a free-living population [102]. In 1990, Ornish et al., reported that a low-fat vegetarian diet (10% calories from fat and 5 mg dietary cholesterol per day) combined with stress management and moderate exercise intervention could cause coronary artery plaque regression [103]. Coronary angiography was repeated 5 years later, and additional regression was found in those maintaining the lifestyle intervention [104].

A subsequent primary prevention study, the multiple risk factor intervention trial (MRFIT) (1974–1982), took those in the top 15% of Framingham risk score and treated hypertension, cigarette smoking, and diet. The dietary intervention was far less strict in terms of cholesterol intake than the Ornish study (5 mg dietary cholesterol per day): 8% saturated fat, 250 mg dietary cholesterol per day, and 10% polyunsaturated fat intake. The dietary goals were not reached, although they did improve from baseline, and the changes in total and LDL cholesterol after 7 years of intervention were modest [105]. Hypertension was better controlled, and smoking was significantly reduced. However, the endpoints of reduction in mortality and coronary death were not achieved [105]. In contrast, a study performed in Spain (2003–2012), the PREvención con DIeta MEDiterránean (PREDIMED) study, demonstrated a 30% reduction in the combined outcomes of stroke, myocardial infarction, and cardiovascular death [106]. In this trial, participants were randomized to 1 of 3 diets: a Mediterranean-style diet supplemented with additional extra virgin olive oil, a Mediterranean-style diet supplemented with mixed nuts, or a control group that was counselled to follow a low-fat diet. Total fat intake was not reduced in those on the Mediterranean diet, whose fat source was fatty fish and plant based. Those randomized to low-fat diet were advised to reduce all types of fat, however adherence was poor, and their intake was similar to baseline diet [106]. The greatest difference in diets between the MRFIT and PREDIMED trials was the percentage intake of monounsaturated fats: 12.7% in the MRFIT and 22.1% and 20.9% in the PREDIMED study. There is now a wealth of literature regarding the cardioprotective effects of the Mediterranean diet’s major constituents: nuts, fruits, vegetables, green leafy vegetables, legumes, whole grain, fish, moderate alcohol, fiber, poultry, and olive oil [106,107,108,109,110,111,112,113,114,115].

In summary, the trials conducted in the 1960s, 1970s, and 1980s focused on reducing saturated fat intake, and while they achieved this and reduced serum cholesterol, they did not reduce incidence of myocardial infarction or coronary artery disease deaths [15]. In contrast, the Diet Reinfarction study [116], the Lyon Heart Study [117], the PREDIMED study [106], and more recent work by Li et al. [115] have shown that constituents of the Mediterranean diet providing healthy monounsaturated fats are more effective in preventing cardiovascular disease and reducing coronary artery disease deaths than low-fat, low-cholesterol diets.

There is also emerging data to suggest that Mediterranean Dietary intervention could be beneficial in heart failure. The alignment of the pathological drivers of heart failure with several beneficial mechanisms of the Mediterranean Diet are highly suggestive [118]. For example, oxidative stress is pathogenic in heart failure [118], and its mediators, such as oxidized low-density lipoprotein and its antibodies, are prognostic [119,120,121]; Mediterranean Dietary intervention reduced markers of inflammation [122]. Tuttolomondo et al., recently described how high-adherence to a Mediterranean-style diet was negatively correlated with presence of heart failure and with heart failure symptom severity, and is consistent with analysis performed by others [123,124]. In a randomized sample of the PREDIMED study, Mediterranean dietary intervention was shown to reduce biomarkers of heart failure and inflammation [122]. Others have described the association between inflammatory markers and diastolic dysfunction in pre-clinical models, the bidirectional relationship of heart failure and inflammation, and suggested that control of inflammation is a critical consideration for the management of heart failure [125,126]. Importantly, Levitan et al., demonstrated that adherence to either the Dietary Approaches to Stop Hypertension (DASH) and Mediterranean dietary patterns reduced heart failure mortality [127].

At the level of dietary components, there are several nutraceuticals present in the Mediterranean diet that have been shown to have beneficial health effects (reviewed in detail recently by Mirabelli et al. [128]). For example, resveratrol, a potent anti-oxidant present in red wine, skin of grapes, berries, and peanuts, has been demonstrated to reduce fasting glycemia and insulinemia, improve insulin sensitivity (reduced homeostatic model assessment of insulin resistance index), and decrease systolic and diastolic blood pressure in patients with type 2 diabetes [129]. Resveratrol acts as a calorie restriction mimetic and activates the sirtuin family of protein deacetylases [130]. Flavonoids are a group of polyphenolic compounds that are abundant in bright-colored fruits and vegetables including cherries, spinach, and citrus fruits. A meta-analysis involving eight prospective studies showed that high intake of flavonoids such as anthocyanidins, flavan-3-ols, flavonols, and isoflavones correlated with reduced risk of type 2 diabetes [131]. Extra virgin olive oil—a key component of the Mediterranean diet—is rich in several bioactive compounds including polyphenols and PUFAs [128]. These compounds have wide-ranging benefits such as attenuation of oxidative stress and improvement of endothelial function via anti-thrombotic and anti-inflammatory mechanisms [132]. This involves inhibiting the adhesion of lymphocytes to vascular endothelium, decreasing the expression of pro-inflammatory cytokines and chemokines, and inhibiting NF-kB signaling [128,132]. Anthocyanins in olive oil exert neuroprotective effects via activation of the peroxisome proliferator-activated receptor γ [128]. The high-mobility group A1 protein (HMGA1) has been described as an important mediator for the positive effects of polyphenols on insulin sensitivity [128]. For example, ferulic acid blocked saturated fat-induced activation of PKC-ε in skeletal muscle cells that led to HMGA1-mediated increase in insulin receptor expression and insulin signaling [133]. Studies in HFD-fed mice showed that administering oleacein—a polyphenolic compound found in olive oil—reduced HFD-induced insulin resistance, adiposity, hepatic steatosis, lipidemia, and inflammatory infiltration of macrophages, and lymphocytes in adipose tissue [134,135]. Oleacein reduced SREBP-1 activation and restored the expression of GLUT 4 in insulin sensitive tissues [134,135]. 

As explained above, there is evidence suggesting that the Mediterranean Diet can be an effective preventative tool for coronary artery disease, metabolic disorders and can also have beneficial effects on heart failure outcomes, which may be at least partly related to anti-inflammatory effects. Data from RCTs such as the PREDIMED RCT suggest that coronary artery disease prevention is mediated through salutary effects on cardiovascular risk factors such as BMI, hypertension, lipids, and diabetes [106]. However, there is now a wealth of data documenting the beneficial effects of Mediterranean Diet, many of which could be orthogonal and additive to mediating traditional risk factors. For example, in Tuttolomondo et al.’s comprehensive review of the topic, in addition to benefits on insulin resistance, hyperinsulinaemia, and blood pressure, they report studies showing upregulation of incretins (e.g., GLP-1), reduction in pro-atherogenic foam cells, reduction of arterial stiffness, reduction in pro-inflammatory ceramides, and increased adiponectin and high-density lipoprotein concentrations [136].

## References

[1] Alwan A. (2011). Global Status Report on Noncommunicable Diseases 2010.

[2] Makinen V.P., Civelek M., Meng Q., Zhang B., Zhu J., Levian C., Huan T., Segre A.V., Ghosh S., Vivar J. (2014). Integrative genomics reveals novel molecular pathways and gene networks for coronary artery disease. PLoS Genet..

[3] NCD Risk Factor Collaboration (NCD-RisC) (2016). Trends in adult body-mass index in 200 countries from 1975 to 2014: A pooled analysis of 1698 population-based measurement studies with 19 2 million participants. Lancet.

[4] Cho N.H., Shaw J.E., Karuranga S., Huang Y., da Rocha Fernandes J.D., Ohlrogge A.W., Malanda B. (2018). IDF Diabetes Atlas: Global estimates of diabetes prevalence for 2017 and projections for 2045. Diabetes Res. Clin. Pract..

[5] Hochberg Z. (2018). An Evolutionary Perspective on the Obesity Epidemic. Trends Endocrinol. Metab..

[6] Elia M., Cummings J. (2007). Physiological aspects of energy metabolism and gastrointestinal effects of carbohydrates. Eur. J. Clin. Nutr..

[7] Eckel R.H., Jakicic J.M., Ard J.D., de Jesus J.M., Houston Miller N., Hubbard V.S., Lee I.M., Lichtenstein A.H., Loria C.M., Millen B.E. (2014). 2013 AHA/ACC guideline on lifestyle management to reduce cardiovascular risk: A report of the American College of Cardiology/American Heart Association Task Force on Practice Guidelines. Circulation.

[8] EFSA Panel on Dietetic Products, Nutrition, and Allergies (NDA) (2010). Scientific Opinion on Dietary Reference Values for fats, including saturated fatty acids, polyunsaturated fatty acids, monounsaturated fatty acids, trans fatty acids, and cholesterol. EFSA J..

[9] Nettleton J.A., Lovegrove J.A., Mensink R.P., Schwab U. (2016). Dietary Fatty Acids: Is it Time to Change the Recommendations?. Ann. Nutr. Metab..

[10] Chiu S., Williams P.T., Krauss R.M. (2017). Effects of a very high saturated fat diet on LDL particles in adults with atherogenic dyslipidemia: A randomized controlled trial. PLoS ONE.

[11] Petersen M.C., Shulman G.I. (2018). Mechanisms of Insulin Action and Insulin Resistance. Physiol. Rev..

[12] Roden M., Shulman G.I. (2019). The integrative biology of type 2 diabetes. Nature.

[13] Smyth S., Heron A. (2006). Diabetes and obesity: The twin epidemics. Nat. Med..

[14] Bray G.A., Popkin B.M. (1998). Dietary fat intake does affect obesity!. Am. J. Clin. Nutr..

[15] Hooper L., Martin N., Jimoh O.F., Kirk C., Foster E., Abdelhamid A.S. (2020). Reduction in saturated fat intake for cardiovascular disease. Cochrane Database Syst. Rev..

[16] de Souza R.J., Mente A., Maroleanu A., Cozma A.I., Ha V., Kishibe T., Uleryk E., Budylowski P., Schunemann H., Beyene J. (2015). Intake of saturated and trans unsaturated fatty acids and risk of all cause mortality, cardiovascular disease, and type 2 diabetes: Systematic review and meta-analysis of observational studies. BMJ.

[17] Chowdhury R., Warnakula S., Kunutsor S., Crowe F., Ward H.A., Johnson L., Franco O.H., Butterworth A.S., Forouhi N.G., Thompson S.G. (2014). Association of dietary, circulating, and supplement fatty acids with coronary risk: A systematic review and meta-analysis. Ann. Intern. Med..

[18] Siri-Tarino P.W., Sun Q., Hu F.B., Krauss R.M. (2010). Meta-analysis of prospective cohort studies evaluating the association of saturated fat with cardiovascular disease. Am. J. Clin. Nutr..

[19] Richard D., Bausero P., Schneider C., Visioli F. (2009). Polyunsaturated fatty acids and cardiovascular disease. Cell. Mol. Life Sci..

[20] Hu F.B. (2010). Are refined carbohydrates worse than saturated fat?. Am. J. Clin. Nutr..

[21] Kroemer G., Lopez-Otin C., Madeo F., de Cabo R. (2018). Carbotoxicity-Noxious Effects of Carbohydrates. Cell.

[22] Ludwig D.S., Willett W.C., Volek J.S., Neuhouser M.L. (2018). Dietary fat: From foe to friend?. Science.

[23] Dehghan M., Mente A., Zhang X., Swaminathan S., Li W., Mohan V., Iqbal R., Kumar R., Wentzel-Viljoen E., Rosengren A. (2017). Associations of fats and carbohydrate intake with cardiovascular disease and mortality in 18 countries from five continents (PURE): A prospective cohort study. Lancet.

[24] Mente A., Dehghan M., Rangarajan S., McQueen M., Dagenais G., Wielgosz A., Lear S., Li W., Chen H., Yi S. (2017). Association of dietary nutrients with blood lipids and blood pressure in 18 countries: A cross-sectional analysis from the PURE study. Lancet Diabetes Endocrinol..

[25] Cao X.-P., Tan C.-C., Yu J.-T. (2018). Associations of fats and carbohydrates with cardiovascular disease and mortality—PURE and simple?. Lancet.

[26] Seidelmann S.B., Claggett B., Cheng S., Henglin M., Shah A., Steffen L.M., Folsom A.R., Rimm E.B., Willett W.C., Solomon S.D. (2018). Dietary carbohydrate intake and mortality: A prospective cohort study and meta-analysis. Lancet Public Health.

[27] Simpson S.J., Raubenheimer D. (2012). The Nature of Nutrition: A Unifying Framework form Animal Adaption to Human Obesity.

[28] Simpson S.J., Batley R., Raubenheimer D. (2003). Geometric analysis of macronutrient intake in humans: The power of protein?. Appetite.

[29] Sorensen A., Mayntz D., Simpson S.J., Raubenheimer D. (2010). Dietary ratio of protein to carbohydrate induces plastic responses in the gastrointestinal tract of mice. J. Comp. Physiol. B.

[30] Solon-Biet S., McMahon A., Ballard J.W.O., Ruohonen K., Wu L., Cogger V., Warren A., Huang X., Pichaud N., Melvin R.G. (2014). The ratio of macronutrients, not caloric intake, dictates cardiometabolic health, aging and longevity in ad libitum-fed mice. Cell Metab..

[31] Gosby A.K., Conigrave A.D., Lau N.S., Iglesias M.A., Hall R.M., Jebb S.A., Brand-Miller J., Caterson I.D., Raubenheimer D., Simpson S.J. (2011). Testing protein leverage in lean humans: A randomised controlled experimental study. PLoS ONE.

[32] Hewson-Hughes A.K., Colyer A., Simpson S.J., Raubenheimer D. (2016). Balancing macronutrient intake in a mammalian carnivore: Disentangling the influences of flavour and nutrition. R. Soc. Open Sci..

[33] Jensen K., Simpson S.J., Nielsen V.H., Hunt J., Raubenheimer D., Mayntz D. (2014). Nutrient-specific compensatory feeding in a mammalian carnivore, the mink, Neovison vison. Br. J. Nutr..

[34] Raubenheimer D., Simpson S.J. (2019). Protein leverage: Theoretical foundations and ten points of clarification. Obesity.

[35] Mayntz D., Raubenheimer D., Salomon M., Toft S., Simpson S.J. (2005). Nutrient-specific foraging in invertebrate predators. Science.

[36] Jensen K., Mayntz D., Toft S., Clissold F.J., Hunt J., Raubenheimer D., Simpson S.J. (2012). Optimal foraging for specific nutrients in predatory beetles. Proc. Biol. Sci..

[37] Simpson S.J., Le Couteur D.G., Raubenheimer D. (2015). Putting the balance back in diet. Cell.

[38] Martinez Steele E., Raubenheimer D., Simpson S.J., Baraldi L.G., Monteiro C.A. (2018). Ultra-processed foods, protein leverage and energy intake in the USA. Public Health Nutr..

[39] Hall K.D., Ayuketah A., Brychta R., Cai H., Cassimatis T., Chen K.Y., Chung S.T., Costa E., Courville A., Darcey V. (2019). Ultra-Processed Diets Cause Excess Calorie Intake and Weight Gain: An Inpatient Randomized Controlled Trial of Ad Libitum Food Intake. Cell Metab..

[40] Buettner R., Scholmerich J., Bollheimer L.C. (2007). High-fat diets: Modeling the metabolic disorders of human obesity in rodents. Obesity.

[41] Fontaine D.A., Davis D.B. (2016). Attention to Background Strain Is Essential for Metabolic Research: C57BL/6 and the International Knockout Mouse Consortium. Diabetes.

[42] Reuter T.Y. (2007). Diet-induced models for obesity and type 2 diabetes. Drug Discov. Today Dis. Model..

[43] Small L., Brandon A.E., Turner N., Cooney G.J. (2018). Modeling insulin resistance in rodents by alterations in diet: What have high-fat and high-calorie diets revealed?. Am. J. Physiol. Endocrinol. Metab..

[44] Nicholson A., Reifsnyder P.C., Malcolm R.D., Lucas C.A., MacGregor G.R., Zhang W., Leiter E.H. (2010). Diet-induced obesity in two C57BL/6 substrains with intact or mutant nicotinamide nucleotide transhydrogenase (Nnt) gene. Obesity.

[45] Hariri N., Thibault L. (2010). High-fat diet-induced obesity in animal models. Nutr. Res. Rev..

[46] Pettersson U.S., Walden T.B., Carlsson P.O., Jansson L., Phillipson M. (2012). Female mice are protected against high-fat diet induced metabolic syndrome and increase the regulatory T cell population in adipose tissue. PLoS ONE.

[47] Burchfield J.G., Kebede M.A., Meoli C.C., Stockli J., Whitworth P.T., Wright A.L., Hoffman N.J., Minard A.Y., Ma X., Krycer J.R. (2018). High dietary fat and sucrose results in an extensive and time-dependent deterioration in health of multiple physiological systems in mice. J. Biol. Chem..

[48] Ishimoto T., Lanaspa M.A., Rivard C.J., Roncal-Jimenez C.A., Orlicky D.J., Cicerchi C., McMahan R.H., Abdelmalek M.F., Rosen H.R., Jackman M.R. (2013). High-fat and high-sucrose (western) diet induces steatohepatitis that is dependent on fructokinase. Hepatology.

[49] Tappy L., Lê K.-A. (2010). Metabolic effects of fructose and the worldwide increase in obesity. Physiol. Rev..

[50] Bortolin R.C., Vargas A.R., Gasparotto J., Chaves P.R., Schnorr C.E., Martinello K.B., Silveira A.K., Rabelo T.K., Gelain D.P., Moreira J.C.F. (2018). A new animal diet based on human Western diet is a robust diet-induced obesity model: Comparison to high-fat and cafeteria diets in term of metabolic and gut microbiota disruption. Int. J. Obes..

[51] Surwit R., Feinglos M., Rodin J., Sutherland A., Petro A., Opara E., Kuhn C., Rebuffe-Scrive M. (1995). Differential effects of fat and sucrose on the development of obesity and diabetes in C57BL/6J and AJ mice. Metabolism.

[52] Farrell G., Schattenberg J.M., Leclercq I., Yeh M.M., Goldin R., Teoh N., Schuppan D. (2019). Mouse Models of Nonalcoholic Steatohepatitis: Toward Optimization of Their Relevance to Human Nonalcoholic Steatohepatitis. Hepatology.

[53] Haeusler R.A., McGraw T.E., Accili D. (2018). Biochemical and cellular properties of insulin receptor signalling. Nat. Rev. Mol. Cell Biol..

[54] DeFronzo R.A., Ferrannini E., Groop L., Henry R.R., Herman W.H., Holst J.J., Hu F.B., Kahn C.R., Raz I., Shulman G.I. (2015). Type 2 diabetes mellitus. Nat. Rev. Dis. Primers.

[55] Brown M.S., Goldstein J.L. (2008). Selective versus total insulin resistance: A pathogenic paradox. Cell Metab..

[56] Perry R.J., Samuel V.T., Petersen K.F., Shulman G.I. (2014). The role of hepatic lipids in hepatic insulin resistance and type 2 diabetes. Nature.

[57] Petersen M.C., Madiraju A.K., Gassaway B.M., Marcel M., Nasiri A.R., Butrico G., Marcucci M.J., Zhang D., Abulizi A., Zhang X.M. (2016). Insulin receptor Thr1160 phosphorylation mediates lipid-induced hepatic insulin resistance. J. Clin. Investig..

[58] Magkos F., Su X., Bradley D., Fabbrini E., Conte C., Eagon J.C., Varela J.E., Brunt E.M., Patterson B.W., Klein S. (2012). Intrahepatic diacylglycerol content is associated with hepatic insulin resistance in obese subjects. Gastroenterology.

[59] Ter Horst K.W., Gilijamse P.W., Versteeg R.I., Ackermans M.T., Nederveen A.J., la Fleur S.E., Romijn J.A., Nieuwdorp M., Zhang D., Samuel V.T. (2017). Hepatic Diacylglycerol-Associated Protein Kinase Cepsilon Translocation Links Hepatic Steatosis to Hepatic Insulin Resistance in Humans. Cell Rep..

[60] Cantley J.L., Yoshimura T., Camporez J.P., Zhang D., Jornayvaz F.R., Kumashiro N., Guebre-Egziabher F., Jurczak M.J., Kahn M., Guigni B.A. (2013). CGI-58 knockdown sequesters diacylglycerols in lipid droplets/ER-preventing diacylglycerol-mediated hepatic insulin resistance. Proc. Natl. Acad. Sci. USA.

[61] Brandon A.E., Liao B.M., Diakanastasis B., Parker B.L., Raddatz K., McManus S.A., O’Reilly L., Kimber E., van der Kraan A.G., Hancock D. (2019). Protein Kinase C Epsilon Deletion in Adipose Tissue, but Not in Liver, Improves Glucose Tolerance. Cell Metab..

[62] Raddatz K., Turner N., Frangioudakis G., Liao B.M., Pedersen D.J., Cantley J., Wilks D., Preston E., Hegarty B.D., Leitges M. (2011). Time-dependent effects of Prkce deletion on glucose homeostasis and hepatic lipid metabolism on dietary lipid oversupply in mice. Diabetologia.

[63] Samuel V.T., Petersen M.C., Gassaway B.M., Vatner D.F., Rinehart J., Shulman G.I. (2019). Considering the Links Between Nonalcoholic Fatty Liver Disease and Insulin Resistance: Revisiting the Role of Protein Kinase C ε. Hepatology.

[64] Morad S.A., Cabot M.C. (2013). Ceramide-orchestrated signalling in cancer cells. Nat. Rev. Cancer.

[65] Holland W.L., Brozinick J.T., Wang L.P., Hawkins E.D., Sargent K.M., Liu Y., Narra K., Hoehn K.L., Knotts T.A., Siesky A. (2007). Inhibition of ceramide synthesis ameliorates glucocorticoid-, saturated-fat-, and obesity-induced insulin resistance. Cell Metab..

[66] Chavez J.A., Summers S.A. (2003). Characterizing the effects of saturated fatty acids on insulin signaling and ceramide and diacylglycerol accumulation in 3T3-L1 adipocytes and C2C12 myotubes. Arch. Biochem. Biophys..

[67] Turpin S.M., Nicholls H.T., Willmes D.M., Mourier A., Brodesser S., Wunderlich C.M., Mauer J., Xu E., Hammerschmidt P., Bronneke H.S. (2014). Obesity-induced CerS6-dependent C16:0 ceramide production promotes weight gain and glucose intolerance. Cell Metab..

[68] Chaurasia B., Tippetts T.S., Mayoral Monibas R., Liu J., Li Y., Wang L., Wilkerson J.L., Sweeney C.R., Pereira R.F., Sumida D.H. (2019). Targeting a ceramide double bond improves insulin resistance and hepatic steatosis. Science.

[69] Stratford S., Hoehn K.L., Liu F., Summers S.A. (2004). Regulation of insulin action by ceramide: Dual mechanisms linking ceramide accumulation to the inhibition of Akt/protein kinase B. J. Biol. Chem..

[70] Powell D.J., Turban S., Gray A., Hajduch E., Hundal H.S. (2004). Intracellular ceramide synthesis and protein kinase Czeta activation play an essential role in palmitate-induced insulin resistance in rat L6 skeletal muscle cells. Biochem. J..

[71] Samuel V.T., Shulman G.I. (2019). Nonalcoholic Fatty Liver Disease, Insulin Resistance, and Ceramides. N. Engl. J. Med..

[72] Bonezzi F., Piccoli M., Dei Cas M., Paroni R., Mingione A., Monasky M.M., Caretti A., Riganti C., Ghidoni R., Pappone C. (2019). Sphingolipid Synthesis Inhibition by Myriocin Administration Enhances Lipid Consumption and Ameliorates Lipid Response to Myocardial Ischemia Reperfusion Injury. Front. Physiol..

[73] Hammerschmidt P., Ostkotte D., Nolte H., Gerl M.J., Jais A., Brunner H.L., Sprenger H.G., Awazawa M., Nicholls H.T., Turpin-Nolan S.M. (2019). CerS6-Derived Sphingolipids Interact with Mff and Promote Mitochondrial Fragmentation in Obesity. Cell.

[74] Osborn O., Olefsky J.M. (2012). The cellular and signaling networks linking the immune system and metabolism in disease. Nat. Med..

[75] Mathis D. (2013). Immunological goings-on in visceral adipose tissue. Cell Metab..

[76] Nishimura S., Manabe I., Nagasaki M., Eto K., Yamashita H., Ohsugi M., Otsu M., Hara K., Ueki K., Sugiura S. (2009). CD8+ effector T cells contribute to macrophage recruitment and adipose tissue inflammation in obesity. Nat. Med..

[77] Hotamisligil G.S., Shargill N.S., Spiegelman B.M. (1993). Adipose expression of tumor necrosis factor-alpha: Direct role in obesity-linked insulin resistance. Science.

[78] Uysal K.T., Wiesbrock S.M., Marino M.W., Hotamisligil G.S. (1997). Protection from obesity-induced insulin resistance in mice lacking TNF-alpha function. Nature.

[79] O’Rourke R.W., Metcalf M.D., White A.E., Madala A., Winters B.R., Maizlin I.I., Jobe B.A., Roberts C.T., Slifka M.K., Marks D.L. (2009). Depot-specific differences in inflammatory mediators and a role for NK cells and IFN-gamma in inflammation in human adipose tissue. Int. J. Obes..

[80] Obstfeld A.E., Sugaru E., Thearle M., Francisco A.M., Gayet C., Ginsberg H.N., Ables E.V., Ferrante A.W. (2010). C-C chemokine receptor 2 (CCR2) regulates the hepatic recruitment of myeloid cells that promote obesity-induced hepatic steatosis. Diabetes.

[81] Lanthier N., Molendi-Coste O., Horsmans Y., van Rooijen N., Cani P.D., Leclercq I.A. (2010). Kupffer cell activation is a causal factor for hepatic insulin resistance. Am. J. Physiol. Gastrointest. Liver Physiol..

[82] Arkan M.C., Hevener A.L., Greten F.R., Maeda S., Li Z.W., Long J.M., Wynshaw-Boris A., Poli G., Olefsky J., Karin M. (2005). IKK-beta links inflammation to obesity-induced insulin resistance. Nat. Med..

[83] de Alvaro C., Teruel T., Hernandez R., Lorenzo M. (2004). Tumor necrosis factor alpha produces insulin resistance in skeletal muscle by activation of inhibitor kappaB kinase in a p38 MAPK-dependent manner. J. Biol. Chem..

[84] Lebrun P., Van Obberghen E. (2008). SOCS proteins causing trouble in insulin action. Acta Physiol..

[85] Samuel V.T., Liu Z.X., Wang A., Beddow S.A., Geisler J.G., Kahn M., Zhang X.M., Monia B.P., Bhanot S., Shulman G.I. (2007). Inhibition of protein kinase Cepsilon prevents hepatic insulin resistance in nonalcoholic fatty liver disease. J. Clin. Investig..

[86] Donath M.Y., Dinarello C.A., Mandrup-Poulsen T. (2019). Targeting innate immune mediators in type 1 and type 2 diabetes. Nat. Rev. Immunol..

[87] Larsen C.M., Faulenbach M., Vaag A., Vølund A., Ehses J.A., Seifert B., Mandrup-Poulsen T., Donath M.Y. (2007). Interleukin-1-receptor antagonist in type 2 diabetes mellitus. N. Engl. J. Med..

[88] van Asseldonk E.J., Stienstra R., Koenen T.B., Joosten L.A., Netea M.G., Tack C.J. (2011). Treatment with Anakinra improves disposition index but not insulin sensitivity in nondiabetic subjects with the metabolic syndrome: A randomized, double-blind, placebo-controlled study. J. Clin. Endocrinol. Metab..

[89] Everett B.M., Donath M.Y., Pradhan A.D., Thuren T., Pais P., Nicolau J.C., Glynn R.J., Libby P., Ridker P.M. (2018). Anti-Inflammatory Therapy With Canakinumab for the Prevention and Management of Diabetes. J. Am. Coll. Cardiol..

[90] Ruscitti P., Masedu F., Alvaro S., Airò P., Battafarano N., Cantarini L., Cantatore F.P., Carlino G., D’Abrosca V., Frassi M. (2019). Anti-interleukin-1 treatment in patients with rheumatoid arthritis and type 2 diabetes (TRACK): A multicentre, open-label, randomised controlled trial. PLoS Med..

[91] Fleischman A., Shoelson S.E., Bernier R., Goldfine A.B. (2008). Salsalate improves glycemia and inflammatory parameters in obese young adults. Diabetes Care.

[92] Shimomura I., Matsuda M., Hammer R.E., Bashmakov Y., Brown M.S., Goldstein J.L. (2000). Decreased IRS-2 and increased SREBP-1c lead to mixed insulin resistance and sensitivity in livers of lipodystrophic and ob/ob mice. Mol. Cell.

[93] Michael M.D., Kulkarni R.N., Postic C., Previs S.F., Shulman G.I., Magnuson M.A., Kahn C.R. (2000). Loss of insulin signaling in hepatocytes leads to severe insulin resistance and progressive hepatic dysfunction. Mol. Cell.

[94] Donnelly K.L., Smith C.I., Schwarzenberg S.J., Jessurun J., Boldt M.D., Parks E.J. (2005). Sources of fatty acids stored in liver and secreted via lipoproteins in patients with nonalcoholic fatty liver disease. J. Clin. Investig..

[95] Abdul-Wahed A., Guilmeau S., Postic C. (2017). Sweet Sixteenth for ChREBP: Established Roles and Future Goals. Cell Metab..

[96] Lustig R.H. (2016). Sickeningly Sweet: Does Sugar Cause Type 2 Diabetes? Yes. Can. J. Diabetes.

[97] AC I. (1908). Influence of animal food on the organism of rabbits. Izvest Imper Voennomed Akad St Petersburg.

[98] Steinberg D. (2004). Thematic review series: The pathogenesis of atherosclerosis. An interpretive history of the cholesterol controversy: Part I. J. Lipid Res..

[99] Ahrens E.H., Hirsch J., Insull W., Tsaltas T.T., Blomstrand R., Peterson M.L. (1957). Dietary control of serum lipids in relation to atherosclerosis. J. Am. Med. Assoc..

[100] Keys A. (1957). Diet and the epidemiology of coronary heart disease. J. Am. Med. Assoc..

[101] Keys A. (1980). Seven Countries: A Multivariate Analysis of Death and Coronary Heart Diseas.

[102] The National Diet-Heart Study Research Group (1968). The National Diet-Heart Study Final Report. Circulation.

[103] Ornish D., Brown S.E., Scherwitz L.W., Billings J.H., Armstrong W.T., Ports T.A., McLanahan S.M., Kirkeeide R.L., Brand R.J., Gould K.L. (1990). Can lifestyle changes reverse coronary heart disease? The Lifestyle Heart Trial. Lancet.

[104] Ornish D., Scherwitz L.W., Billings J.H., Brown S.E., Gould K.L., Merritt T.A., Sparler S., Armstrong W.T., Ports T.A., Kirkeeide R.L. (1998). Intensive lifestyle changes for reversal of coronary heart disease. JAMA.

[105] Multiple Risk Factor Intervention Trial Research Group (1982). Multiple risk factor intervention trial. Risk factor changes and mortality results. Multiple Risk Factor Intervention Trial Research Group. JAMA.

[106] Estruch R., Ros E., Salas-Salvado J., Covas M.I., Corella D., Aros F., Gomez-Gracia E., Ruiz-Gutierrez V., Fiol M., Lapetra J. (2013). Primary prevention of cardiovascular disease with a Mediterranean diet. N. Engl. J. Med..

[107] Dehghan M., Mente A., Teo K.K., Gao P., Sleight P., Dagenais G., Avezum A., Probstfield J.L., Dans T., Yusuf S. (2012). Relationship between healthy diet and risk of cardiovascular disease among patients on drug therapies for secondary prevention: A prospective cohort study of 31 546 high-risk individuals from 40 countries. Circulation.

[108] Trichopoulou A., Costacou T., Bamia C., Trichopoulos D. (2003). Adherence to a Mediterranean diet and survival in a Greek population. N. Engl. J. Med..

[109] Mente A., de Koning L., Shannon H.S., Anand S.S. (2009). A systematic review of the evidence supporting a causal link between dietary factors and coronary heart disease. Arch. Intern. Med..

[110] Rimm E.B. (2002). Fruit and vegetables-building a solid foundation. Am. J. Clin. Nutr..

[111] Iqbal R., Anand S., Ounpuu S., Islam S., Zhang X., Rangarajan S., Chifamba J., Al-Hinai A., Keltai M., Yusuf S. (2008). Dietary patterns and the risk of acute myocardial infarction in 52 countries: Results of the INTERHEART study. Circulation.

[112] Bazzano L.A., He J., Ogden L.G., Loria C.M., Vupputuri S., Myers L., Whelton P.K. (2002). Fruit and vegetable intake and risk of cardiovascular disease in US adults: The first National Health and Nutrition Examination Survey Epidemiologic Follow-up Study. Am. J. Clin. Nutr..

[113] Joshipura K.J., Hu F.B., Manson J.E., Stampfer M.J., Rimm E.B., Speizer F.E., Colditz G., Ascherio A., Rosner B., Spiegelman D. (2001). The effect of fruit and vegetable intake on risk for coronary heart disease. Ann. Intern. Med..

[114] Hu F.B., Rimm E.B., Stampfer M.J., Ascherio A., Spiegelman D., Willett W.C. (2000). Prospective study of major dietary patterns and risk of coronary heart disease in men. Am. J. Clin. Nutr..

[115] Li S., Chiuve S.E., Flint A., Pai J.K., Forman J.P., Hu F.B., Willett W.C., Mukamal K.J., Rimm E.B. (2013). Better diet quality and decreased mortality among myocardial infarction survivors. JAMA Intern. Med..

[116] Burr M.L., Fehily A.M., Gilbert J.F., Rogers S., Holliday R.M., Sweetnam P.M., Elwood P.C., Deadman N.M. (1989). Effects of changes in fat, fish, and fibre intakes on death and myocardial reinfarction: Diet and reinfarction trial (DART). Lancet.

[117] de Lorgeril M., Renaud S., Mamelle N., Salen P., Martin J.L., Monjaud I., Guidollet J., Touboul P., Delaye J. (1994). Mediterranean alpha-linolenic acid-rich diet in secondary prevention of coronary heart disease. Lancet.

[118] Tuttolomondo A., Di Raimondo D., Casuccio A., Velardo M., Salamone G., Cataldi M., Corpora F., Restivo V., Pecoraro R., Della Corte V. (2020). Mediterranean diet adherence and congestive heart failure: Relationship with clinical severity and ischemic pathogenesis. Nutrition.

[119] Metra M., Dinatolo E., Dasseni N. (2019). The New Heart Failure Association Definition of Advanced Heart Failure. Card. Fail. Rev..

[120] Tsutsui H., Kinugawa S., Matsushima S. (2011). Oxidative stress and heart failure. Am. J. Physiol. Heart Circ. Physiol..

[121] Tsutsui T., Tsutamoto T., Wada A., Maeda K., Mabuchi N., Hayashi M., Ohnishi M., Kinoshita M. (2002). Plasma oxidized low-density lipoprotein as a prognostic predictor in patients with chronic congestive heart failure. J. Am. Coll. Cardiol..

[122] Fito M., Estruch R., Salas-Salvado J., Martinez-Gonzalez M.A., Aros F., Vila J., Corella D., Diaz O., Saez G., de la Torre R. (2014). Effect of the Mediterranean diet on heart failure biomarkers: A randomized sample from the PREDIMED trial. Eur. J. Heart Fail..

[123] Tektonidis T.G., Akesson A., Gigante B., Wolk A., Larsson S.C. (2015). A Mediterranean diet and risk of myocardial infarction, heart failure and stroke: A population-based cohort study. Atherosclerosis.

[124] Tektonidis T.G., Akesson A., Gigante B., Wolk A., Larsson S.C. (2016). Adherence to a Mediterranean diet is associated with reduced risk of heart failure in men. Eur. J. Heart Fail..

[125] Sartori M., Conti F.F., Dias D.D.S., Dos Santos F., Machi J.F., Palomino Z., Casarini D.E., Rodrigues B., De Angelis K., Irigoyen M.C. (2017). Association between Diastolic Dysfunction with Inflammation and Oxidative Stress in Females ob/ob Mice. Front. Physiol..

[126] Van Linthout S., Tschope C. (2017). Inflammation-Cause or Consequence of Heart Failure or Both?. Curr. Heart Fail. Rep..

[127] Levitan E.B., Lewis C.E., Tinker L.F., Eaton C.B., Ahmed A., Manson J.E., Snetselaar L.G., Martin L.W., Trevisan M., Howard B.V. (2013). Mediterranean and DASH diet scores and mortality in women with heart failure: The Women’s Health Initiative. Circ. Heart Fail..

[128] Mirabelli M., Chiefari E., Arcidiacono B., Corigliano D.M., Brunetti F.S., Maggisano V., Russo D., Foti D.P., Brunetti A. (2020). Mediterranean Diet Nutrients to Turn the Tide against Insulin Resistance and Related Diseases. Nutrients.

[129] Zhu X., Wu C., Qiu S., Yuan X., Li L. (2017). Effects of resveratrol on glucose control and insulin sensitivity in subjects with type 2 diabetes: Systematic review and meta-analysis. Nutr. Metab..

[130] Wahl D., Bernier M., Simpson S.J., de Cabo R., Le Couteur D.G. (2018). Future directions of resveratrol research. Nutr. Healthy Aging.

[131] Xu H., Luo J., Huang J., Wen Q. (2018). Flavonoids intake and risk of type 2 diabetes mellitus: A meta-analysis of prospective cohort studies. Medicine.

[132] Summerhill V., Karagodin V., Grechko A., Myasoedova V., Orekhov A. (2018). Vasculoprotective Role of Olive Oil Compounds via Modulation of Oxidative Stress in Atherosclerosis. Front. Cardiovasc. Med..

[133] Gogoi B., Chatterjee P., Mukherjee S., Buragohain A.K., Bhattacharya S., Dasgupta S. (2014). A polyphenol rescues lipid induced insulin resistance in skeletal muscle cells and adipocytes. Biochem. Biophys. Res. Commun..

[134] Lombardo G.E., Lepore S.M., Morittu V.M., Arcidiacono B., Colica C., Procopio A., Maggisano V., Bulotta S., Costa N., Mignogna C. (2018). Effects of Oleacein on High-Fat Diet-Dependent Steatosis, Weight Gain, and Insulin Resistance in Mice. Front. Endocrinol..

[135] Lepore S.M., Maggisano V., Bulotta S., Mignogna C., Arcidiacono B., Procopio A., Brunetti A., Russo D., Celano M. (2019). Oleacein Prevents High Fat Diet-Induced Adiposity and Ameliorates Some Biochemical Parameters of Insulin Sensitivity in Mice. Nutrients.

[136] Tuttolomondo A., Simonetta I., Daidone M., Mogavero A., Ortello A., Pinto A. (2019). Metabolic and Vascular Effect of the Mediterranean Diet. Int. J. Mol. Sci..

